# Factors influencing the contamination rates of the conjunctival swabs and organ culture media of human donor eyes

**DOI:** 10.1097/MD.0000000000011879

**Published:** 2018-09-21

**Authors:** Tobias Röck, Johanna Landenberger, Michael Buhl, Efdal Yoeruek, Karl Ulrich Bartz-Schmidt, Matthias Bramkamp, Gunnar Blumenstock, Daniel Röck

**Affiliations:** a Centre for Ophthalmology; b Institute of Medical Microbiology and Hygiene, University of Tübingen, Tübingen; c Department of General Medicine, Ruhr-University Bochum, Bochum; d Institute for Clinical Epidemiology and Applied Biometry, University of Tübingen, Tübingen, Germany.

**Keywords:** bacteria, conjunctival swab, contamination, cornea, fungi, microbiological testing

## Abstract

This study assessed the influence of donor, environmental, and logistical factors on the contamination rates of the conjunctival swabs and organ culture media of human donor eyes.

In total, 1008 conjunctival swabs and 418 organ culture media samples from 504 consecutive human donor eyes were analyzed. Cross-tabulation, chi-squared tests, and Fisher's exact tests were used to assess the influence of the different factors on the contamination rates of the conjunctival swabs and organ culture media.

The overall contamination rates were 28.4% for the conjunctival swabs and 1.0% for the organ culture media. A prolonged time between death and the conjunctival swab collection was associated with an increased conjunctival swab contamination rate [odds ratio (OR) = 1.9, 95% confidence interval (CI) = 1.2–3.0, *P = *.007]. The highest conjunctival swab contamination rate was found in the corneas procured in external institutions (outside the university hospital) (44.1%, OR = 3.6, 95%CI = 1.5–8.4, *P = *.003). Hospitalization times of 2 to 7 days prior to death were associated with an increased conjunctival swab contamination risk (OR = 2.6, 95%CI = 1.1–5.8, *P = *.021). However, the sex, age, cause of donor death, differentiation between septic and aseptic donors, differentiation between heart-beating brain-dead *multiorgan donors* and cadaveric donors, a warmer mean monthly temperature, and death to corneoscleral disc excision time did not significantly increase the conjunctival swab contamination risk. In addition, none of these factors affected the organ culture media contamination risk. Moreover, a positive conjunctival swab did not significantly increase the media contamination risk (*P = *.08). Surprisingly, the microorganisms causing media contamination were present at 50% of the amount detected on the conjunctival surface of the respective donor eye.

A prolonged time between death and the conjunctival swab collection, a hospitalization time of 2 to 7 days prior to death, and corneal collection outside the university hospital seemed to be the main factors responsible for an increased conjunctival swab contamination risk. In addition, our investigation illustrated that a positive conjunctival swab is not a strong indicator for organ culture media contamination. Critical discussion is necessary regarding the validity of conjunctival swabs as prognostic parameters for organ culture media contamination.

## 1 Introduction

In addition to poor endothelial quality and medical contraindications, such as a serology reactive or nonevaluable donor status, corneal contamination is the third leading cause of discarding donor corneas.^[1–3]^

The transplantation of contaminated corneal tissue may lead to postoperative endophthalmitis, which represents one of the most serious post-keratoplasty complications, and it may have devastating consequences for a patient's vision.^[4–9]^ Contamination rates of 0.53% to 15.7% have been reported^[10–21]^; therefore, extensive decontamination procedures have been advocated to minimize the risk of donor cornea contamination. Until January 2018, the German guidelines on the collection and processing of donor corneas have required 2 microbiological examinations of the donor's cornea using conjunctival swabs after disinfection and before the corneal excision or enucleation.^[22,23]^ In spite of donor eye decontamination with povidone-iodine, persistent microorganismal colonization has been demonstrated.^[24–27]^

In this study, we investigated the influence and correlations of several factors on and between the contamination of conjunctival swabs and organ culture media, like sex, donor age, cause of death, heart-beating brain-dead *multiorgan donor* versus cadaveric donor, septic donor versus aseptic donor, hospitalization time prior to death, procurement site, death to conjunctival swab collection time, enucleation to processing time, and mean monthly temperature.

To the best of our knowledge, the evaluation of so many factors on the contamination of conjunctival swabs and organ culture media has never been undertaken before. Nor have the correlations been determined between the microbial growth in the conjunctival swabs of corneal donors and organ culture media contamination in an eye bank, during a study period without changing standards, while simultaneously considering the microbiological species.

## 2 Methods

### 2.1 Eye donors

In the period from July 2015 to September 2017, 504 corneas from 252 consecutive donors were stored at the Tübingen Eye Bank in Tübingen, Germany. No maximum donor age limit was set, and the minimum donor age was 14 years old. In 2016, our study group showed that our collective would have lost nearly 14% of the grafts collected for transplantation by using a maximum donor age (>79 years old).^[2]^ We came to the conclusion that older donors cannot generally be excluded from corneal donation due to the scarcity of grafts available for keratoplasties in Germany.

A detailed medical history of each donor was obtained from interviews with the family, the last attending doctor, the donor's family doctor, and a review of any hospital medical records. The consent and medical history of each donor were recorded. The donor serology was analyzed from a blood sample taken up to 24 hours postmortem.

Blood samples had been drawn for the mandatory infectious disease tests, including human immunodeficiency virus (HIV), hepatitis B virus (HBV), hepatitis C virus (HCV), and syphilis. The infectious disease testing was done as per previously published methods.^[1]^ A culture period beginning up to 72 hours postmortem was accepted. The beginning of the culture period was defined as the time when the prepared and excised corneoscleral discs were first placed in the tissue culture flasks (Corning Incorporated, New York, NY) filled with culture medium (Culture Medium I; Biochrom GmbH, Berlin, Germany) with 2.5% fetal bovine serum (FBS; Merck Millipore, Darmstadt, Germany). Any potential donors with high-risk sexual behaviors or intravenous drug use, and consequently, at high risk for any of the infectious pathogens (HIV, HBV, or HCV) were not eligible to donate.

This study was approved by the institutional review board of the University of Tübingen, and it adhered to the tenets of the Declaration of Helsinki.

### 2.2 Decontamination, swab collection, enucleation, preparation, and corneal storage

The periocular region (lids, brows, and cheeks) and the ocular surface (cornea, conjunctiva, and palpebral fornices) were cleaned using a 0.75% povidone-iodine solution [1 mL of 7.5% Braunol (B. Braun Melsungen AG, Melsungen, Germany) diluted with 10 mL of sterile 0.9% NaCl (B. Braun Melsungen, AG)] for at least 3 minutes. The face and the head of the donor were covered with sterile surgical drapes, leaving the eye region open. The medical doctor performing the procedure wore a sterile gown, sterile gloves, a surgical cap, and a mask. Before enucleation, 2 conjunctival swabs (from the upper and lower conjunctival fornices) were taken from each eye (BBL CultureSwab Plus; Becton Dickinson, Franklin Lakes, NJ). All corneas were procured by members of the Tübingen eye bank which means that the swabs and samples taken in external institutions were taken by the same doctors taking the samples in the principal institutions. After a limbal-based conjunctival incision, a 360° peritomy was performed. Following the blunt dissection of the muscles from the conjunctiva and Tenon's capsule, the muscles were transected using a muscle hook and scissors. The optic nerve was cut with curved blunt scissors to allow a complete enucleation.

The 2 globes were stored separately in sterile urine cups (Sarstedt, Numbrecht, Germany). We placed each eye in gauze at the bottom of the sterile cup, which was filled with 10 mL of 0.9% physiological NaCl (B. Braun Melsungen AG) and 5 mL of gentamicin eye drops (Merck Pharma GmbH, Darmstadt, Germany). The eyes were transported to our eye bank on a cool pack in a cool box (temperature between 1°C and 10°C). Here, the donor globes were stored in a refrigerator at 4°C until the preparation of the corneoscleral discs in a class II biological safety cabinet. Before preparing the corneoscleral discs, all excess conjunctiva was removed from the donor globes, and they were immersed separately in a 0.375% povidone-iodine solution [2.5 mL of 7.5% Braunol diluted with 50 mL of sterile 0.9% NaCl (B. Braun Melsungen AG)] for 5 min, followed by rinsing with 50 mL of sterile 0.9% NaCl (B. Braun Melsungen AG). Then, a 15-mm trephine was used to cut the sclera around the cornea, and the cut was completed using scissors. The corneoscleral disc was carefully separated from the iris and the uveal tissue using scissors and tweezers. After that, the corneoscleral disc was placed on a corneal holder (Bausch & Lomb, Heidelberg, Germany) and put into a tissue culture flask (Corning Incorporated) filled with Culture Medium I (Biochrom AG) with 2.5% FBS (Merck Millipore). Culture Medium I is supplemented with 60 μg/mL of penicillin G sodium, 130 μg/mL of streptomycin sulfate, and 2.5 μg/mL of amphotericin B. The corneoscleral discs were stored in an incubator at 37°C, 5% CO_2_, and 95% humidity. Sterile instruments were used throughout the whole process, including the enucleation, corneoscleral disc excision, and endothelial examination. The processing of each whole globe was performed in a class II biological safety cabinet by one experienced technician.

### 2.3 Microbiological testing

#### 2.3.1 Conjunctival swabs

The conjunctival swabs were transported and stored at room temperature. All the samples were tested immediately at the Institute of Medical Microbiology and Hygiene (University Hospital of Tübingen). Supplemented Columbia sheep blood agar plates (Oxoid GmbH, Wesel, Germany), Endo agar plates, and supplemented brain heart infusion (BHI) agar plates (Institute of Medical Microbiology and Hygiene) were incubated at 37°C to test for bacterial contamination. A liver broth was also used. Additionally, yeast-gentamicin plates (Institute of Medical Microbiology and Hygiene) were used to detect fungal contamination, and they were incubated at 30°C. The plates were incubated for 10 days. They were r*ead* after 24 and 48 hours *and after 10 days.*

If there was cultural growth, the microorganisms were detected by matrix-assisted laser desorption/ionization time-of-flight mass spectrometry (Microflex; Bruker Corporation, Billerica, MA). *Any proof of bacterial or fungal microorganisms was documented as contamination.*

#### 2.3.2 Organ culture media

The culture flasks underwent daily visual inspections for turbidity. After 3 days of incubation in the Tübingen Eye Bank the culture media were routinely tested for contamination. For this, each corneal medium was exchanged in a class II biological safety cabinet, and it was tested for bacterial and fungal contamination at the Institute of Medical Microbiology and Hygiene. The supplemented Columbia sheep blood agar plates (Oxoid GmbH), Endo agar plates, and supplemented BHI agar plates (Institute of Medical Microbiology and Hygiene) were incubated at 37°C to test for bacterial contamination. A liver broth was also used. Yeast-gentamicin plates (Institute of Medical Microbiology and Hygiene) were used to detect fungal contamination, and they were incubated at 30°C. The plates were incubated for 7 days, and they were r*ead* after 24 and 48 hours *and after 7 days*. The contaminated corneas were removed from the eye bank. Two days before transplantation, the culture medium was exchanged and retested for contamination. *Any proof of bacterial or fungal microorganisms was documented as contamination.*

### 2.4 Evaluation

We collected the relevant donor and storage data, including the sex, donor age, cause of death, heart-beating brain-dead *multiorgan donor* versus cadaveric donor, septic donor versus aseptic donor, hospitalization time prior to death, procurement site, death to conjunctival swab collection time, enucleation to processing time, and the mean monthly temperature. To explore whether the seasonal temperature changes corresponded to the contamination rates of the conjunctival swabs and organ culture media, the donation months were divided into 2 separate groups: May through September, which included the warmer months, and October through April, which included the cooler months.

The mean monthly temperature data of Stuttgart-Echterdingen, which is close to Tübingen, was obtained from the German meteorological service homepage.^[28]^ There is no weather station in Tübingen; therefore, we used the data from the next closest weather station (Stuttgart-Echterdingen). The *distance as the crow flies* is around 20 kilometers.

### 2.5 Statistical analysis

The statistical analysis of the data was conducted using IBM SPSS Statistics for Windows, version 24.0 (IBM Corp., Armonk, NY). The categorical data were analyzed using cross-tabulations and Pearson's chi-squared tests. Fisher's exact tests were used as tests of association, as appropriate. The quantitative data were reported as the mean with the standard deviation. The odds ratios (ORs) were quoted with 95% confidence intervals (CIs), and *P* < .05 was considered to be statistically significant.

## 3 Results

This retrospective study included the microbiological testing of 1008 conjunctival swabs and 418 organ culture media samples from 504 consecutive donor eyes. Around 86 corneas and their culture media samples were discarded before the 3 days of incubation were over. The main reasons therefore were serology-reactive or nonevaluable blood samples of the donors. The male-to-female ratio was 62% to 38%. The overall contamination rate of the conjunctival swabs was 28.4%, and that of the organ culture media was 1.0%. The mean donor age was 68 ± 15 years old (range 17–96 years). The most common causes of death were cardiovascular disease (34.8%), infection (14.4%), cancer (12.4%), and multiple organ dysfunction syndrome (12.4%). The mean endothelial cell density at beginning of the storage time was 2261 ± 490 cells/mm^2^. The mean time between death and the conjunctival swab collection was 12.4 ± 15.0 hours, and the mean time between death and the corneoscleral disc excision was 27.3 ± 12.3 hours. The time period between death and the conjunctival swab collection was divided into 2 groups: group 1 was < 6 hours and group 2 was 6 to 72 hours. The contamination rates of the conjunctival swabs were 20.9% in group one and 33.1% in group 2 (Table 1). A prolonged time between death and the conjunctival swab collection was significantly associated with an increased conjunctival swab contamination rate (OR = 1.9, 95%CI = 1.2–3.0, *P = *.007). The organ culture media contamination rates were 1.6% in group 1 and 0.7% in group 2. The time interval between death and the conjunctival swab collection had no statistically significant influence on the contamination rate (*P = *.6).

**Table 1:**
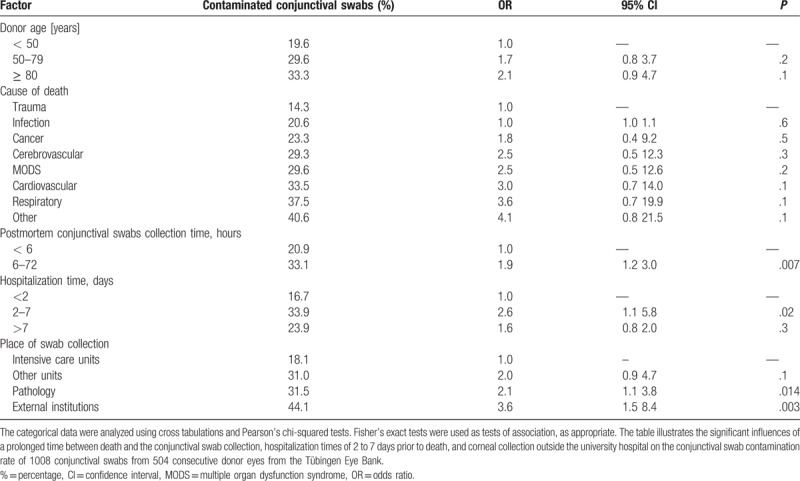
Factors influencing the contamination risk of donor eye conjunctival swabs.

The 4 swab collection places were distinguished between the intensive care unit of the University Hospital Tübingen, the other units of the University Hospital Tübingen, the pathology and cold room of the University Hospital Tübingen, and external institutions. With regard to the procurement sites, the highest conjunctival swab contamination rate was found in the corneas procured in external institutions (funeral institutes or departments outside the university hospital) (44.1%, OR = 3.6, 95%CI = 1.5–8.4, *P = *.003). The second highest conjunctival swab contamination rate was found in the corneas procured in the pathology department of the University Hospital Tübingen (OR = 2.08, 95%CI = 1.1–3.8, *P = *.014) (Table 1).

Hospitalization times of 2 to 7 days were associated with an increased risk of conjunctival swab contamination (OR = 2.6, 95%CI = 1.1–5.8, *P = *.021) (Table 1). However, the following factors had no statistically significant influence on the conjunctival swab and organ culture media contamination rates: sex (*P = *.4 and *P* = 1.0, respectively), cause of donor death (0.1≤p≤0.6 and *P* = 1.0, respectively), donor age (*P = *.2, *P = *.1, and *P* = 1.0, respectively), heart-beating brain-dead *multiorgan donor* versus cadaveric donor (*P = *.5 and *P* = 1.0, respectively), septic donor versus aseptic donor (*P = *.2 and *P = *.6, respectively), and mean monthly temperature of the warmer months (*P = *.9 and *P* = 1.0, respectively).

### 3.1 Contaminating organisms

A total of 95% of the conjunctival swab contamination organisms were of bacterial origin. The most common bacteria belonging to the normal skin bacterial flora (70.7%), including coagulase negative staphylococci, *Corynebacterium* spp. and *Streptococcus* spp., but excluding *Streptococcus pneumoniae*. The most common bacteria not belonging to the normal flora were *Staphylococcus aureus* (8.2%), *Enterococcus* spp. (3.8%), and *Escherichia coli* (3.3%). Around 5% of the conjunctival swab contamination organisms were of fungal origin, exclusively *Candida* spp. Around 75% of the organ culture media contamination organisms were of fungal origin, exclusively *Candida* spp., and 25% were of bacterial origin, exclusively gram-positive organisms. There is no apparent common factor with the patients that gave positive fungal results for the culture media contamination.

The microorganisms causing the medium contamination were a mere 50% equivalent to those detected on the conjunctival surface of the respective donor eye.

## 4 Discussion

Our investigation illustrates that a prolonged time between death and the conjunctival swab collection, hospitalization times of 2 to 7 days prior to death, and corneal collection outside the university hospital seemed to be mainly responsible for the increased conjunctival swab contamination risk in 1008 conjunctival swabs from 504 consecutive donor eyes at the Tübingen Eye Bank. The different factors evaluated did not have an effect on the 8 organ culture media samples tested, and a positive conjunctival swab did not significantly increase the medium contamination risk.

The positive conjunctival swab rate of 28.4% found in our investigation can be placed in the range of the 7.0% to 86.2% positive swabs found in previous studies.^[29–32]^ This wide range may be due to the varying decontamination protocols and different conjunctival swab origins. While Reddy and Paul found a 7.0% positive rate in patients admitted for cataract surgery (eyes in a living state-control group) after inserting one drop of proparacaine into the conjunctival sack,^[29]^ Mindrup et al, Matsumoto et al, and Fuest et al^[30–32]^ obtained rates in line with our results of 28.9%, 36.7%, and 22.8%, respectively, after disinfection with povidone-iodine in corneal donor cadavers. Without decontamination before the swab collection, Capriotti et al^[33]^ obtained a rate of 86.2%.

*Staphylococcus* spp., *Corynebacterium* spp., and *Streptococcus* spp. (excluding *Streptococcus pneumonia*) were the predominant microorganisms found on the conjunctival swabs in our study, which fits the bacterial spectrum of the human conjunctiva described by Matsumoto et al, Reddy and Paul, Fuest et al, and Wilhelm et al.^[29,31,32,34]^

Donor eye decontamination before graft processing plays a significant role in preventing tissue contamination. Many years ago, povidone-iodine was known to be one of the best antiseptic solutions as a skin preparation for the reduction of microorganisms. In 1982, the bactericidal activity of various concentrations of povidone-iodine was tested by Berkelman et al.^[35]^ They demonstrated that low concentrations (i.e., 0.1%–1%) were more rapidly bactericidal than a full strength treatment (i.e., 10% solution).

The main reasons for the very low organ culture media contamination rate in our investigation in contrast to other studies could be the different graft preparations and processing. Decontamination protocols and decontamination procedures vary in many ways. In our study, the periocular region, the fornices, and the ocular surface were cleaned using a 0.75% povidone-iodine solution for at least 3 minutes. After sterile enucleation, the donor globes were also immersed separately in a 0.375% povidone-iodine solution for 5 min. In accordance with our study, Laubichler et al^[36]^ recommended the use of a 0.75% povidone-iodine solution for at least 3 minutes to decontaminate the donor eyes. However, in total, we decontaminated the donor eyes for at least 8 minutes in low concentrations of povidone-iodine and transported the eyes separately between the 2 decontaminations in sterile cups filled with 5 mL of gentamicin eye drops.

Our extensive decontamination protocol, as described above, could explain why our organ culture media contamination rate of 1.0% was lower than the rates of 4.5% to 10.8% found by Li et al,^[27]^ who cleaned the periocular region with a 7.5% povidone-iodine solution spray and the ocular surface with a 1.25% povidone-iodine solution for 3 and 5 minutes, respectively. Furthermore Li et al abstained from the use of gentamicin, and they prepared the corneoscleral disc directly at the donor site instead of in a class II biological safety cabinet, without decontaminating twice, as in our study. Despite the slightly similar steps, no complete execution of the entire detailed decontamination protocol described in our investigation could be found in any of the other studies considered.

With regard to the hospitalization times of more than 7 days prior to death, no significantly increased conjunctival swab contamination risk was seen. The reason for this might be that these patients were typically submitted to extensive systemic antibiotic and antimycotic treatments before death. For this reason, the microbiological flora in the conjunctival compartment may also have been reduced.

Contrary to our investigation not discovering any factors influencing the culture media contamination rate, Armitage et al^[37]^ determined that cancer, infections, and respiratory diseases as causes of death significantly increased the culture media contamination risk. The fact that there was no significant factor influencing the culture media contamination rate in this study was probably due to the low contamination rate of 1%.

Because the microorganisms causing the culture media contamination were tested as sensitive to several antibiotic and antifungal substances in our study, the addition of different antibiotics and antifungals to the culture medium can be discussed in the future. The use of voriconazole, as a triazole reacting differently than the currently used polyene amphotericin, is conceivable. The aminoglycoside streptomycin and beta-lactam penicillin could be supplemented by the fluoroquinolone levofloxacin in the future. Both levofloxacin and voriconazole have been described as nontoxic to corneal endothelial cells.^[38,39]^ However, this should be tested in vitro on human corneas over a longer culture period of time before routine use.

Nevertheless, some points should be considered before drawing hasty conclusions. The main limitation of our evaluation was the pilot nature of the observations. Studies in the future will require a larger sample size, which would increase the power of the analysis and the validity of its findings. Another limiting factor of this study was the limited comparability of these data with other publications on the results of the microbiological testing of conjunctival swabs and organ culture media. The other studies used different methods of excising the corneoscleral disc only versus enucleating the entire globe, different decontamination protocols at different concentrations and repetitions, and different methods of microbiological testing. In future studies the investigation on antibiotic and antimycotic treatments before donor death and its influence on the contamination rates of the conjunctival swabs could be considered as well as the addition of different antibiotics and antifungals to the culture medium. To increase the comparability with other publications on this topic the microbiological testing could distinguish between different germs instead of summarizing several bacteria as normal skin bacterial flora.

In conclusion, this investigation illustrates that a prolonged time between death and the conjunctival swab collection, hospitalization times of 2 to 7 days prior to death, and corneal collection outside the university hospital seemed to be the main factors responsible for an increased conjunctival swab contamination risk. Surprisingly, the microorganisms causing the medium contamination were only 50% equivalent to the organisms detected on the conjunctival surface of the respective donor eye. Moreover, a positive conjunctival swab was not a strong indicator for corneal culture medium contamination. Therefore, a critical discussion is necessary about the validity of conjunctival swabs as prognostic parameters for organ culture media contamination.

## Author contributions

**Conceptualization:** Tobias Röck, Gunnar Blumenstock.

**Data curation:** Tobias Röck, Johanna Landenberger, Daniel Röck.

**Formal analysis:** Tobias Röck, Johanna Landenberger.

**Investigation:** Tobias Röck, Johanna Landenberger, Michael Buhl, Efdal Yoeruek, Daniel Röck.

**Methodology:** Tobias Röck, Johanna Landenberger, Efdal Yoeruek, Karl Ulrich Bartz-Schmidt, Matthias Bramkamp, Gunnar Blumenstock, Daniel Röck.

**Project administration:** Tobias Röck, Daniel Röck.

**Resources:** Michael Buhl, Efdal Yoeruek.

**Software:** Johanna Landenberger, Gunnar Blumenstock.

**Supervision:** Tobias Röck, Karl Ulrich Bartz-Schmidt, Matthias Bramkamp, Gunnar Blumenstock, Daniel Röck.

**Validation:** Tobias Röck, Johanna Landenberger, Michael Buhl, Matthias Bramkamp, Gunnar Blumenstock, Daniel Röck.

**Visualization:** Tobias Röck, Gunnar Blumenstock.

**Writing – original draft:** Tobias Röck, Daniel Röck.

**Writing – review & editing:** Tobias Röck, Michael Buhl, Efdal Yoeruek, Karl Ulrich Bartz-Schmidt, Matthias Bramkamp, Gunnar Blumenstock.
