# Factors influencing the contamination rates of the conjunctival swabs and organ culture media of human donor eyes: Erratum

**DOI:** 10.1097/MD.0000000000012852

**Published:** 2018-10-12

In the article, “Factors influencing the contamination rates of the conjunctival swabs and organ culture media of human donor eyes”,^[1]^ which appears in Volume 97, Issue 38 of *Medicine*, the sentence “The different factors evaluated did not have an effect on the 8 organ culture media samples tested, and a positive conjunctival swab did not significantly increase the medium contamination risk” on page 4 should appear as “The different factors evaluated did not have an effect on the organ culture media samples tested, and a positive conjunctival swab did not significantly increase the medium contamination risk.”
